# Supplementary material for: Pseudomolecule-scale genome assemblies of Drepanocaryum sewerzowii and Marmoritis complanata
Source: G3 (Bethesda). 2024 Jul 24;14(10):jkae172. doi: 10.1093/g3journal/jkae172 (PMC11979756; doi:10.1093/g3journal/jkae172)
Supplement: jkae172_Supplementary_Data [file jkae172_supplementary_data.zip › Figure_S3_G3-2024-405070.pdf]

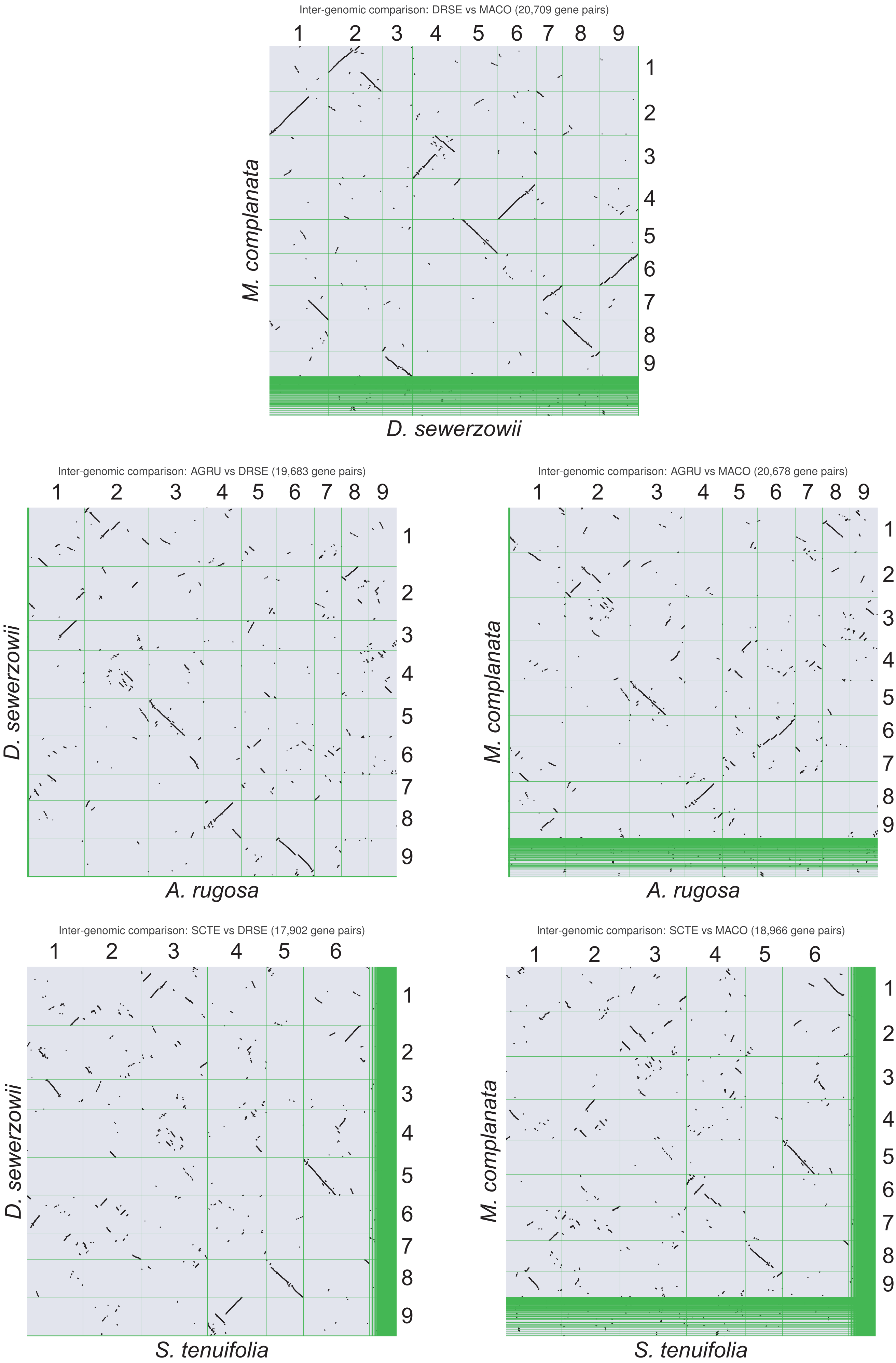
Inter-genomic comparison: DRSE vs MACO (20,709 gene pairs)
M. complanata
D. sewerzowii
Inter-genomic comparison: AGRU vs DRSE (19,683 gene pairs)
D. sewerzowii
A. rugosa
Inter-genomic comparison: AGRU vs MACO (20,678 gene pairs)
M. complanata
A. rugosa
Inter-genomic comparison: SCTE vs DRSE (17,902 gene pairs)
D. sewerzowii
S. tenuifolia
Inter-genomic comparison: SCTE vs MACO (18,966 gene pairs)
M. complanata
S. tenuifolia
