# Supplementary material for: Pseudomolecule-scale genome assemblies of Drepanocaryum sewerzowii and Marmoritis complanata
Source: G3 (Bethesda). 2024 Jul 24;14(10):jkae172. doi: 10.1093/g3journal/jkae172 (PMC11979756; doi:10.1093/g3journal/jkae172)
Supplement: jkae172_Supplementary_Data [file jkae172_supplementary_data.zip › Figure_S4_G3-2024-405070.pdf]

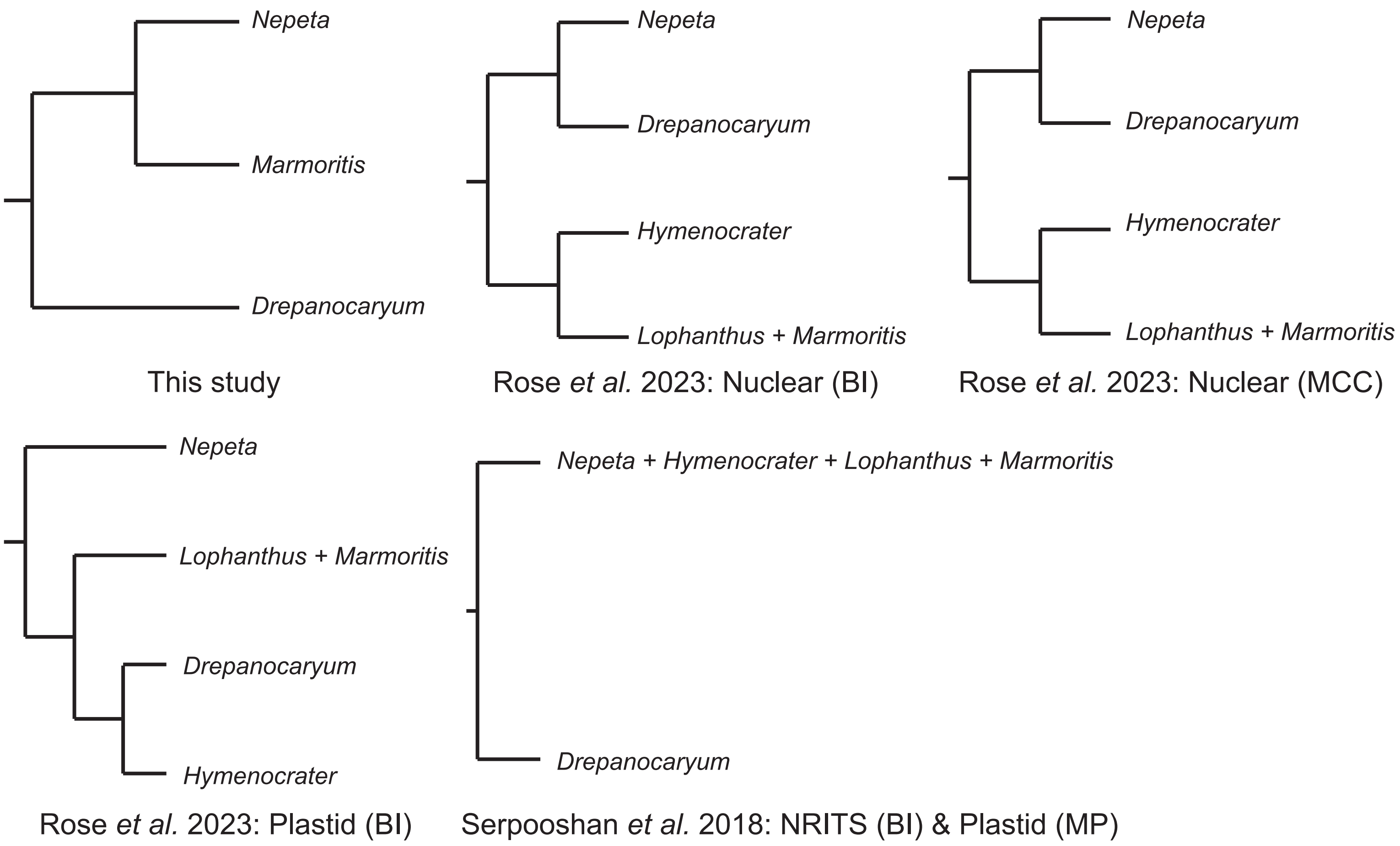
Nepeta
Marmoritis
Drepanocaryum
This study
Nepeta
Drepanocaryum
Hymenocrater
Lophanthus + Marmoritis
Rose et al. 2023: Nuclear (BI)
Nepeta
Drepanocaryum
Hymenocrater
Lophanthus + Marmoritis
Rose et al. 2023: Nuclear (MCC)
Nepeta
Lophanthus + Marmoritis
Drepanocaryum
Hymenocrater
Rose et al. 2023: Plastid (BI)
Nepeta + Hymenocrater + Lophanthus + Marmoritis
Drepanocaryum
Serpooshan et al. 2018: NRITS (BI) & Plastid (MP)
