# Supplementary material for: Pseudomolecule-scale genome assemblies of Drepanocaryum sewerzowii and Marmoritis complanata
Source: G3 (Bethesda). 2024 Jul 24;14(10):jkae172. doi: 10.1093/g3journal/jkae172 (PMC11979756; doi:10.1093/g3journal/jkae172)
Supplement: jkae172_Supplementary_Data [file jkae172_supplementary_data.zip › Supplemental_Figure_Legends_G3-2024-405070.docx]

**Supplementary Figure Legends**

**Figure S1.** FCM ungated histograms showing the distribution of stained nuclei for *D. sewerzowii* (A) and *M. complanata* (B). *N. cataria* (G1 NECA) and *N. racemosa* (G1 NEMU) references are shown relative to that of *D. sewerzowii* and *M. complanata*, labelled as G1 sample. Genomescope K-mer estimation of genome size and heterozygosity is shown for *D. sewerzowii* (C) and *M. complanata* (D) with a heterozygosity estimation of 0.0839% and 0.586%, respectively.

**Figure S2.** HiC contact maps for *D. sewerzowii* (A) and *M. complanata* (B). Green boxes show contigs orientated to chromosomes (blue boxes).

**Figure S3.** Pairwise dot plots illustrating collinear regions between *D. sewerzowii*, *M. complanata*, *A. rugosa* and *S. tenuifolia*. Numbers on the axes show the chromosome number.

**Figure S4.** Summary of tree incongruence between the genome derived species tree (this study) and trees reported using Bayesian Inference (BI), maximum clade credibility (MCC) or maximum parsimony (MP) for tree inference with plastid markers, nuclear markers or nuclear ribosomal internal transcribed spacer regions (NRITS).
