# Supplementary material for: Pseudomolecule-scale genome assemblies of Drepanocaryum sewerzowii and Marmoritis complanata
Source: G3 (Bethesda). 2024 Jul 24;14(10):jkae172. doi: 10.1093/g3journal/jkae172 (PMC11979756; doi:10.1093/g3journal/jkae172)
Supplement: jkae172_Supplementary_Data [file jkae172_supplementary_data.zip › Table_S1_G3-2024-405070.docx]

| **Table S1 - Genome assemblies used for comparative genomics and phylogenomics.** | |
| --- | --- |
| **Species** | **Reference** |
| *Agastache rugosa* (Fisch. & C.A.Mey.) Kuntze | [Park *et al.* (2023)](https://paperpile.com/c/Nm3Ym6/ZOtqS) |
| *Callicarpa americana* L. | [Hamilton *et al.* (2020)](https://paperpile.com/c/Nm3Ym6/hicOs) |
| *Drepanocaryum sewerzowii* (Regel) Pojark. | This work |
| *Hyssopus officinalis* L. | [Lichman *et al.* (2020)](https://paperpile.com/c/Nm3Ym6/r1BlE) |
| *Isodon rubescens* (Hemsl.) H.Hara | [Sun *et al.* (2023)](https://paperpile.com/c/Nm3Ym6/6O9Xr) |
| *Lavandula angustifolia* Mill. | [Hamilton *et al.* (2023)](https://paperpile.com/c/Nm3Ym6/8oRE9) |
| *Marmoritis complanata* (Dunn) A.L.Budantzev | This work |
| *Mentha longifolia* (L.) L. | [Vining *et al.* (2022)](https://paperpile.com/c/Nm3Ym6/zVjee) |
| *Nepeta cataria* L. | [Lichman *et al.* (2020)](https://paperpile.com/c/Nm3Ym6/r1BlE) |
| *Nepeta racemosa* Lam. | [Lichman *et al.* (2020)](https://paperpile.com/c/Nm3Ym6/r1BlE) |
| *Ocimum basilicum* L. | [Bornowski *et al.* (2020)](https://paperpile.com/c/Nm3Ym6/tufNc) |
| *Origanum majorana* L. | [Bornowski *et al.* (2020)](https://paperpile.com/c/Nm3Ym6/tufNc) |
| *Origanum vulgare* L. | [Bornowski *et al.* (2020)](https://paperpile.com/c/Nm3Ym6/tufNc) |
| *Paulownia fortunei* (Seem.) Hemsl. | [Cao *et al.* (2021)](https://paperpile.com/c/Nm3Ym6/JBlZ2) |
| *Perilla citriodora* (Makino) Nakai | [Zhang *et al.* (2021)](https://paperpile.com/c/Nm3Ym6/jmbHC) |
| *Perilla frutescens* (L.) Britton | [Zhang *et al.* (2021)](https://paperpile.com/c/Nm3Ym6/jmbHC) |
| *Pogostemon cablin* (Blanco) Benth. | [Shen *et al.* (2022)](https://paperpile.com/c/Nm3Ym6/GybYj) |
| *Salvia bowleyana* Dunn | [Zheng *et al.* (2021)](https://paperpile.com/c/Nm3Ym6/cxfUW) |
| *Salvia hispanica* L. | [Wang *et al.* (2022)](https://paperpile.com/c/Nm3Ym6/0dpx0) |
| *Salvia miltiorrhiza* Bunge | [Pan *et al.* (2023)](https://paperpile.com/c/Nm3Ym6/AygUo) |
| *Salvia rosmarinus* Spenn. | [Han *et al.* (2023)](https://paperpile.com/c/Nm3Ym6/UWm7D) |
| *Salvia splendens* Sellow ex Nees | [Jia *et al.* (2021)](https://paperpile.com/c/Nm3Ym6/EZvB0) |
| *Schizonepeta tenuifolia* (Benth.) Briq. | [Liu *et al.* (2023)](https://paperpile.com/c/Nm3Ym6/rLry5) |
| *Scutellaria baicalensis* Georgi | [Xu *et al.* (2020)](https://paperpile.com/c/Nm3Ym6/kypF8) |
| *Scutellaria barbata* D.Don | [Xu *et al.* (2020)](https://paperpile.com/c/Nm3Ym6/kypF8) |
| *Teucrium marum* L. | [Smit *et al.* (2024)](https://paperpile.com/c/Nm3Ym6/QJulF) |
| *Thymus quinquecostatus* Čelak. | [Sun *et al.* (2022)](https://paperpile.com/c/Nm3Ym6/7EZgi) |
