# Supplementary figures and images for: Pseudomolecule-scale genome assemblies of Drepanocaryum sewerzowii and Marmoritis complanata
Source: G3 (Bethesda). 2024 Jul 24;14(10):jkae172. doi: 10.1093/g3journal/jkae172 (PMC11979756; doi:10.1093/g3journal/jkae172)

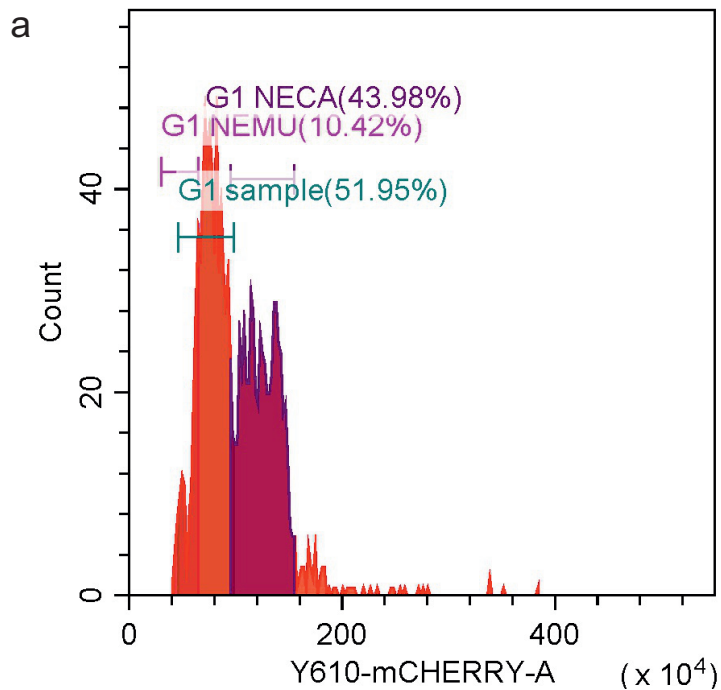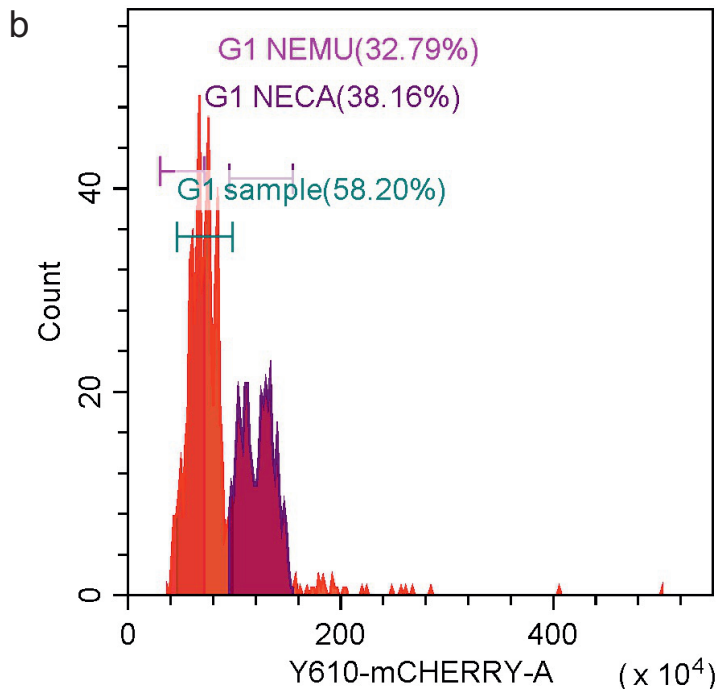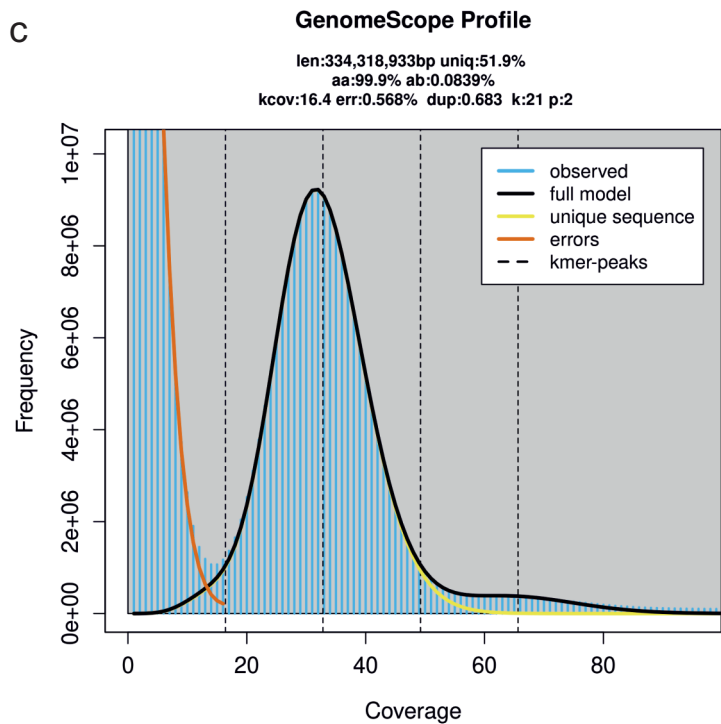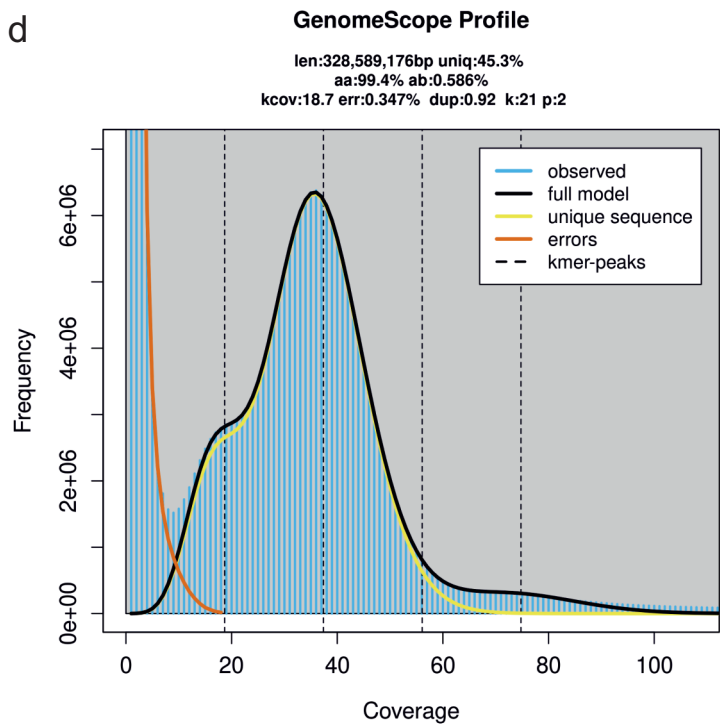

Supplement: jkae172_Supplementary_Data [file jkae172_supplementary_data.zip › Figure_S1_G3-2024-405070.pdf]

a

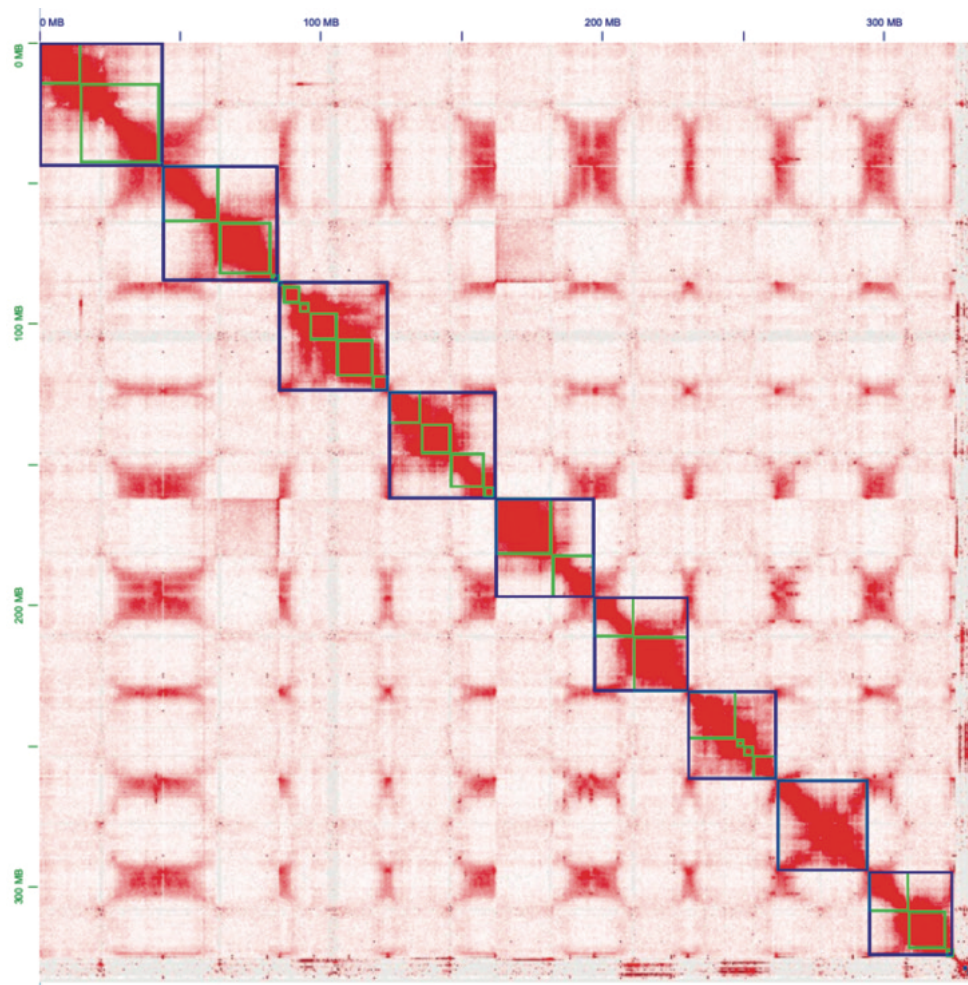

b

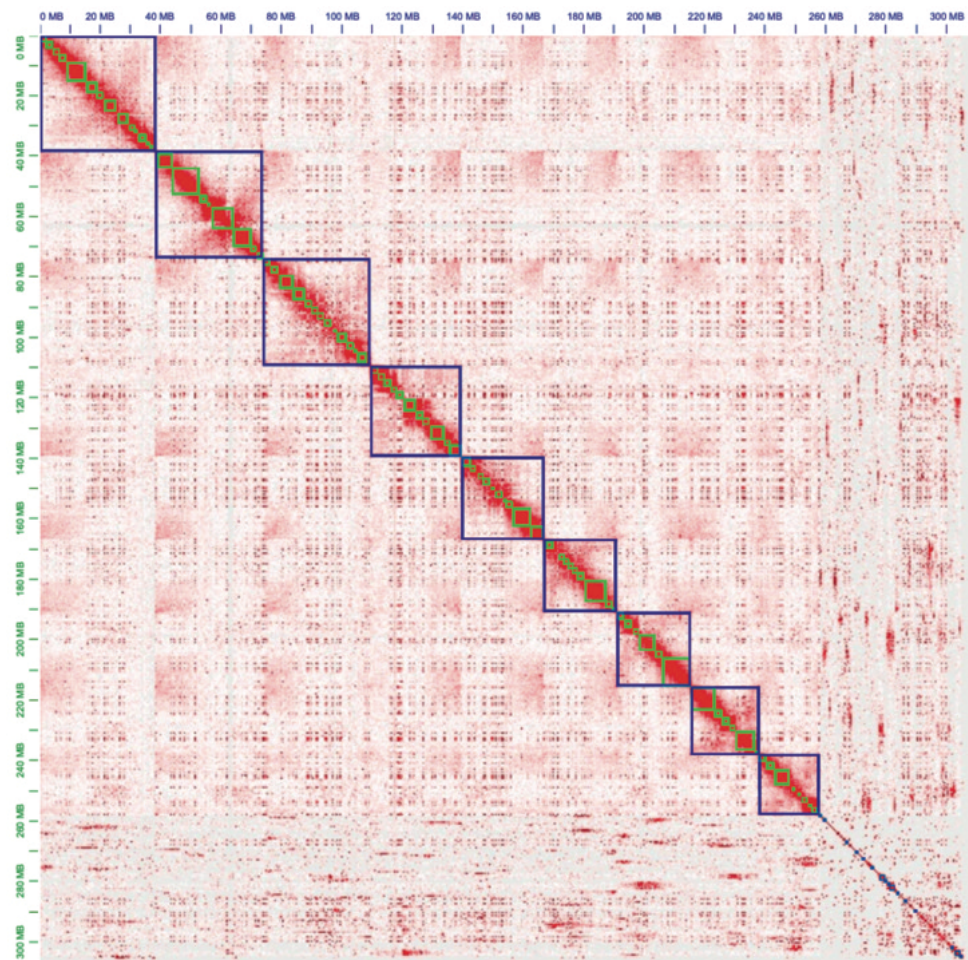

Supplement: jkae172_Supplementary_Data [file jkae172_supplementary_data.zip › Figure_S2_G3-2024-405070.pdf]
